# Drug-resistant tuberculosis: is India ready for the challenge?

**DOI:** 10.1136/bmjgh-2018-000971

**Published:** 2018-08-10

**Authors:** Soumya Chatterjee, Husain Poonawala, Yogesh Jain

**Affiliations:** 1 Division of Infectious Diseases, Allergy and Immunology, Saint Louis University, St Louis, Missouri, USA; 2 Department of Immunology, National Institute for Research in Tuberculosis, Chennai, Tamil Nadu, India; 3 Jan Swasthya Sahyog, Ganiyari, Chhattisgarh, India

**Keywords:** tuberculosis, health systems, public health, epidemiology, health policy

**Summary box**

- India contributes to one-fourth of the global burden of multidrug-resistant tuberculosis (MDR-TB) with inadequate diagnostic infrastructure for drug susceptibility testing (DST).
- A survey of anti-TB drug-resistance demonstrates high rates of resistance to first-line and second-line antitubercular drugs in new and previously treated cases of TB in India.
- The survey is likely underestimating the burden of antitubercular drug resistance in India.
- India needs more laboratories to meet the goal of universal DST.
- A multipronged strategy investing in laboratory capacity, addressing isoniazid monoresistance, designing empiric MDR-TB regimens, involving the private sector and improving airborne infection control will be necessary to bring drug-resistant TB under control.

## Introduction

Tuberculosis (TB) kills close to half a million Indians every year.^1^ Additionally, a million ‘missing’ undiagnosed or inadequately diagnosed cases go unnotified annually.^2^ Not surprisingly, drug-resistant tuberculosis (DR-TB) is a significant problem, and India now has the most number of cases of multidrug-resistant tuberculosis (MDR)-TB in the world, contributing one-fourth of the global burden.^1^ The treatment of MDR-TB requires the use of toxic drugs, is long and expensive and has substantially lower success rates than for drug-sensitive TB.^1^ In this commentary, we review the burden of drug resistance in India considering recent data from India^3^ and discuss areas of focus necessary to combat DR-TB.

## What is the burden of DR-TB in India?

Globally, 4.1% of new TB cases are reported to be MDR.^1^ Concordant with previous surveys, the First National Anti-Tuberculosis Drug Resistance survey conducted by the Indian Government in collaboration with the World Health Organization (WHO) and the United States Agency for International Development (USAID) showed that close to 23% of new cases have resistance to any drug with MDR-TB detected in 3%.^3^ Monoresistance to rifampin was not seen and resistance to isoniazid (INH) was highest (any 11%, monoresistance 4%), followed by resistance to pyrazinamide (any 7%, monoresistance 4%) and streptomycin (any 7%, monoresistance 2%).^3^ Among patients previously treated for TB, there were high levels of resistance to first-line drugs—tested isoniazid (any 25%, monoresistance 8%) followed by resistance to streptomycin (any 13%, monoresistance 2%), pyrazinamide (any 9%, monoresistance 4%) and ethambutol (any 7%, monoresistance 0.21%).^3^

Fluoroquinolones (FQ) are essential components of DR-TB regimens, and FQ resistance can lead to the development of extensively DR-TB (XDR-TB).^4^ Most recent surveys from India have reported rates of FQ resistance close to 21% in non-MDR patients and 36% among those with MDR-TB.^4^ In the resistance survey, equivalent or higher rates of FQ resistance (24%) were noted in new patients with TB with MDR-TB compared with previously treated TB cases (21%).^3^ India suffers from rampant empiric FQ use (including over-the-counter purchase) for a wide range of infections, compounded by poor regulatory capacity leading to widespread availability of counterfeit preparations.

Additionally, the survey demonstrated that 7% of new patients with TB and 2% of previously treated patients were resistant to the aminoglycosides amikacin, capreomycin or kanamycin, which with FQs are vital drugs in the treatment of MDR-TB. Resistance to ethionamide and para-amino-salicylic acid were 11% each in newly treated patients and 7% and 4%, respectively, in previously treated patients. XDR-TB was found in 1.3% of surveyed samples.

The high rates of drug resistance to both first-line and second-line drugs is alarming, but it is likely the survey is underestimating the true burden of resistance in India. The survey only included patients with smear-positive TB, excluding smear-negative TB and extrapulmonary TB, as well as patients diagnosed in jails and prisons. Most striking was the lack of involvement of the private sector, which may be treating more cases of TB than is currently estimated, with high rates of drug resistance reported from cities like Mumbai.^5 6^

The approximately 5300 patients that were sampled represent less than 0.2% of the 2.8 million annual cases of TB. Additionally, the 120 TB units sampled cover 1.3%–2.5% of the entire population; since the distribution of drug resistance in the country is not uniform, this sampling strategy may have excluded areas with high rates of DR-TB. This is reflected by the absence of primary MDR-TB cases in Haryana, Jammu and Kashmir, Karnataka, Meghalaya, Orissa and Telangana in the survey,^3^ whereas there were 3264 cases of MDR-TB cases reported to the government in 2017 as per the India Report 2018.^7^ Between 2015 and 2017, our hospital in Chhattisgarh, in rural central India, obtained phenotypic drug-susceptibility testing (DST) using Lowenstein-Jensen (LJ) media in 417 samples and found 8% of treatment-naive patients to have INH monoresistance and 3.5% to have MDR-TB.

## Laboratory capacity for diagnosis of DR-TB in India

Phenotypic DST for TB is performed using solid culture (LJ) or liquid culture (MGIT 960), both of which require time (2–12 weeks), resources and expertise and hence are performed only in referral laboratories. Molecular assays such as Xpert MTB/RIF (Gene Xpert) and line probe assays (LPAs) detect resistance in hours compared with the weeks required for phenotypic DSTs. The Revised National Tuberculosis Control Programme (RNTCP) recommends that DST be performed only in those with a history of previous treatment for TB, or for those with risk factors for resistance. Under the National Strategic Programme for Tuberculosis 2017–2025 (NSP),^8^ the government intends to perform DST (phenotypic or molecular) on all TB samples, but at present universal DST is performed on samples from 257 of 712 districts in the country.^7^

India currently has 628 Gene Xpert machines and 74 RNTCP certified laboratories to perform susceptibility testing.^7^ In 2017, under the RNTCP, India performed 1.07 million Xpert MTB/RIF tests, 93 989 LPA tests, and second-line DST for 26 832 samples.^7^ However, to diagnose the estimated 2.8 million cases of TB and 150 000 cases of MDR-TB every year, the number of laboratories and the number of samples tested in each laboratory will need to be scaled up.

Commercial molecular tests detect select resistance-conferring mutations and are currently unavailable for many second-line drugs. Whole-genome sequencing (WGS) studies from India have demonstrated novel mutations that may not be detected by commercial tests.^9^ In addition, with 238 mutations across 18 genetic loci responsible for resistance to 13 first-line and second-line drugs,^10^ detection of resistance to drugs other than rifampin may not be amenable to a simple molecular test. TB strains circulating in India differ from those in other parts of the world,^9^ and commercial tests may perform differently in India than they do elsewhere. In these situations, phenotypic DST may be the only way to detect resistance, for which building phenotypic DST capacity is vital.

WGS offers great potential for the rapid and comprehensive diagnosis of resistance and is becoming routine for the identification of anti-TB drug resistance in the developed world. India is building the capacity to perform WGS at all six National TB Referral Laboratories. Investments in bioinformatics and multidisciplinary studies to determine significance of novel mutations will be necessary for WGS to impact clinical care.^11^

Ultimately, the goal should be to detect all cases of TB and DR-TB through universal culture and DST and develop the capacity to perform targeted sequencing or WGS to detect novel mutations.

## Is INH monoresistance being taken seriously enough?

Nearly 90% of INH resistance in India is caused by *KatG* mutations, associated with high-level resistance and poor treatment outcomes^12^; the development of INH resistance precedes the development of MDR-TB.^13^ Initial INH resistance increases incidence rates of treatment failure and relapse compared with pan-sensitive strains (incidence rate ratio 10.9 and 1.8, respectively).^14^ Data from the most recent National workshop on DST-guided treatment in India reveals poor treatment success rates for INH monoresistant TB, ranging from 31% to 53%.^2^ Studies will need to define clinical risk factors for INH monoresistance, perform universal DST to allow detection of INH resistance in all cases, and conduct prospective trials to determine optimal treatment regimens for patients with INH monoresistance.

## Empiric treatment of MDR-TB and contacts

Indian guidelines recommend treating previously treated TB cases as presumptive DR-TB. However, phenotypic DST results may take months, and the results of the current survey present the challenge in selecting an appropriate treatment regimen of 4–5 active drugs for previously treated TB cases given the delays in obtaining DST results. With high rates of FQ and streptomycin resistance, the best empiric regimen until DST results is unknown. Additionally, it is unclear what prophylactic regimens should be used for contacts of patients with MDR-TB. Further research to design empiric regimens for both these populations is essential.

## Involvement of the private sector

An estimated 1 million cases of TB are not reported to the government every year, and the majority of these are believed to be in the private sector; the actual numbers may be 2–3 times higher.^5^ Additionally, the quality of TB care in the private sector is suboptimal with wide variations in knowledge and adherence to guidelines, increasing the risk of development of drug resistance.^15^ While TB was made a notifiable disease in 2012, fewer than 40% of cases from the private sector were notified to the RNTCP in 2017.^7^ Improving case notifications rates from the private sector and ensuring that patients receive high-quality care will be necessary to co DR-TB.

## Airborne transmission and infection control

Contrary to the widespread belief that previous treatment is a major risk factor for MDR-TB, recent studies suggest that most MDR-TB is transmitted rather than acquired, accounting for 96% of new and 61% of previously treated cases of MDR-TB.^16^ Modelling studies estimate that 85% of TB in India in 2032 will be MDR-TB, all due to primary transmission.^17^ Healthcare facilities in India have poor airborne infection control systems, with only 10% of healthcare workers wearing N95 masks.^18^ This is reflected in the high prevalence of latent and active TB among healthcare workers in India.^19 20^ WGS studies have demonstrated transmission of TB between patients in healthcare facilities in India, suggesting they could serve as sites for spread of DR-TB.^9^ Improvements in infection control will reduce the transmission of TB (especially MDR-TB) within healthcare facilities. Identifying transmission networks in the community using traditional and molecular epidemiological methods will be necessary to locate hotspots that require targeted interventions.

## Conclusion

India has set an ambitious goal of TB elimination by 2025. The large burden of DR-TB will limit progress towards that goal. Rarely, does the cliché ‘prevention is better than cure’ carry as much weight as it does with DR-TB. We believe that a multipronged strategy focusing on improving diagnostic capacity, guaranteeing high-quality treatment and preventing transmission will be central to meeting the challenge of DR-TB in India.
